# Secondary Preventive Care for Cardiovascular Diseases in Bangladesh: A National Survey

**DOI:** 10.5334/gh.953

**Published:** 2021-04-30

**Authors:** Sayed Ibn Alam, Jamal Uddin, Fakhrul Islam Khaled, Harisul Hoque, Dipak K. Adhikary, Rezaul Karim, M. A. Rashid, Sajal Krishna Banerjee, Rod S. Taylor, Ann-Dorthe Olsen Zwisler, Sherry L. Grace

**Affiliations:** 1 REHPA – Danish Knowledge Centre for Rehabilitation and Palliative Care, Odense University Hospital and University of Southern Denmark, Nyborg, DK; 2 Physiotherapy and Cardiac Rehabilitation Unit, Ibrahim Cardiac Hospital and Research Institute, Dhaka, BD; 3 Department of Cardiology, University Cardiac Center, Bangabandhu Sheikh Mojib Medical University, Dhaka, BD; 4 Department of Cardiology, Ibrahim Cardiac Hospital and Research Institute, Dhaka, BD; 5 MRC/CSO Social and Public Health Sciences Unit & Robertson Centre for Biostatistics, Institute of Health and Wellbeing, University of Glasgow, Glasgow, GB; 6 York University & KITE-University Health Network, University of Toronto, CA

**Keywords:** Cardiac rehabilitation, secondary prevention, low- and middle-income countries, health services, health policy

The prevalence of cardiovascular diseases (CVD) is increasing worldwide, particularly in low- and middle-income countries (LMICs) [1]. Due to lack of preventive care and screening for CVD risk factors, people in LMICs often develop CVD at a younger age and have poorer outcomes [2]. CVD is major cause of disability in the LMIC of Bangladesh for example, accounting for 13% of all disability-adjusted life years (DALYs) lost [3].

The International Council of Cardiovascular Prevention and Rehabilitation (ICCPR) performed an audit of cardiac rehabilitation (CR) availability globally in 2017. It identified only one program in Bangladesh [4], despite over 400,000 incident ischemic heart disease cases annually [3]. While acute care for CVD including percutaneous coronary interventions and coronary artery bypass graft surgery is available in Bangladesh both in the public and private sectors [5], it is unfortunate such life-saving [6] and cost-efficient chronic care services are not.

To augment CR capacity, there is a need to identify the cardiac centers in Bangladesh that could deliver it, and to understand what secondary prevention is currently being offered. To our best knowledge, no study has yet established this. Therefore, the purpose of this study was to establish the availability and extent of cardiac secondary prevention services in Bangladesh.

Data for this cross-sectional study was collected between August and October 2019 using Epidata. A list of all medical colleges, institutes and specialized cardiac hospitals both in the government and private sectors was collated from the Bangladesh Medical and Dental Council website [7] and through contacts of the authors. The inclusion criterion was that the center has a cardiology/cardiac surgery department; centers with only neonatal cardiac departments were excluded.

Cardiologists, cardiovascular surgeons, or other physicians treating cardiac patients from eligible centers were contacted about the survey by phone call. Where there was a positive response, a consent form and questionnaire were (e)mailed, or a face-to-face interview was performed where possible, based on preference. Non-responders were contacted via email and/or phone. An alternative physician was contacted from the same center if a response was not received within a further two weeks.

Survey items were used from ICCPR’s global CR program survey [8], assessing the nature of the clinical center, CVD risk factors assessed, as well as secondary prevention components delivered. Descriptive statistics were calculated using STATA 16.1.

As shown in Supplemental Figure 1, there were 106 centers, of which 79.2% were successfully contacted. Nineteen did not meet inclusion criteria (so 77.4% of the potential centers did, suggesting there could be approximately 106 * 0.774 = 82 acute cardiac centers in the country). The response rate was 80%.

Respondents were predominantly physicians (cardiologist, cardiac surgeon, registrar). Centers were mostly located in urban (n = 32; 62.7%) and suburban (n = 18; 35.2%) areas. In the capital city Dhaka, there were 26 centers that offered cardiac services, but in 14 out 20 districts, there was no specialized cardiac care (Supplemental Figure 2). In 98% of centers (n = 50), patients pay out-of-pocket for their care. No centers had electronic patient records.

In terms of risk factor assessment (Supplemental Table 1), blood pressure, tobacco use, cholesterol, and diabetes assessment were undertaken by most of the centers. Physical inactivity, diet, and anthropometric assessment was undertaken by ≥80%. However, anxiety and depression assessment were less frequent, with sleep apnea least commonly assessed.

For secondary preventive care (Table 1), most centers offered management of identified risk factors and nutrition counseling. Many offered psychological counselling. Smoking cessation treatment was scantly available.

**Table 1:** Cardiovascular secondary prevention services/care delivered by Bangladesh cardiology centers.

	n	%

Individual consultation with a physician	51	100.0
Individual consultation with a nurse	0	0.0
Exercise stress test	34	66.6
Other functional capacity test	7	13.7
Assessment of strength (e.g., handgrip)	9	17.6
Assessment for comorbidities	42	82.3
Exercise prescription	2	3.9
Physical activity counseling	50	98.0
Supervised exercise training	1	1.9
Heart rate measurement training/exercise intensity assessment	1	1.9
Resistance training	0	0.0
Management of cardiovascular risk factors	48	94.1
Prescription and/or titration of secondary prevention medications	43	84.3
Nutrition counseling	48	94.1
Psychological counseling	43	84.3
Stress management/Relaxation techniques	31	60.7
Smoking cessation sessions/classes	3	5.8
Vocational counseling/support for return-to-work	19	37.2
Communication of patient assessment results with their primary care provider	0	0.0
Follow-up after as outpatient	41	80.3
Other	0	0.0

Our national survey of all medical college hospitals, institutes and specialized hospitals reveals grossly insufficient acute and chronic CV care capacity in Bangladesh [9]. About half of all centers were located in greater Dhaka (capital city), with 70% of districts having no specialized CVD care. Reported rates of screening for the main CV risk factors was quite high, but use of structured exercise was low. Furthermore, although consumption of tobacco is high in Bangladesh [10] and its use is associated with greater mortality in CVD, smoking cessation interventions were offered infrequently.

The clinical and policy implications of this audit of secondary preventive care are many. It is clear that availability of CVD secondary preventive care needs to be augmented in Bangladesh, starting first with regions where none exists. Results confirmed there remains only one CR program in Bangladesh. If we assume based on ICCPR’s audit that each CR program could treat 400 patients/year [4], and given the incidence of CVD in Bangladesh [3], approximately 1,000 more programs would be needed across the country. Overcoming barriers at the healthcare system, healthcare professional, and patient (i.e., accessibility, affordability) levels is necessary. Efforts to increase the number of CR programs could include approaching local philanthropists, professional bodies, and government to introduce legislation. Indeed, our group, comprised of multiple stakeholders, is working to secure funding to validate a cross-cultural adaptation of technology-based CR established as effective in higher-resource settings [11]. ICCPR has a training certification program to augment CR human resource capacity (https://globalcardiacrehab.com/Certification), which has been successfully implemented across India (1000 physicians). This course is suitable for multiple professions involved in CVD secondary prevention, and given there was little consultation with nurses, this may be an important area to start [2].

Limitations of this study must be acknowledged. The survey was conducted in English, which is the language used in medical school, but not the first language spoken in the country. Second, self-reported data is prone to error; we did not visit each center to verify responses or verify data from a second healthcare professional at each institution. Finally, we did not assess CVD care that may be assessed in outpatient settings, community, or primary care.

In conclusion, despite availability of advanced cardiac care technology, CV secondary prevention is insufficiently available both in government and private hospitals in Bangladesh. Most districts/divisions had no specialized cardiac care, as most were located in the capital Dhaka. While the available acute cardiac centers assessed the main risk factors, there is a need to deliver more tobacco cessation interventions. There remains only one CR program in the entire country. We must advocate to policymakers for CR to reduce the CVD burden in Bangladesh.

## Data Accessibility Statement

Data used in the research project have not been made available because this was not outlined at the time of research ethics submission, and respondents were not asked to consent to make the data available online. Moreover, given the small sample sizes in most regions, privacy could be breached. However, the corresponding author will consider reasonable requests for data from established investigators with appropriate approvals, to provide the data in a non-identifiable manner.

## Additional Files

The additional files for this article can be found as follows:

10.5334/gh.953.s1 Supplemental Figure 1. Flow diagram depicting identification of cardiac centres and response rate.

10.5334/gh.953.s2 Supplemental Figure 2. Responding center location by Bangladesh district.

10.5334/gh.953.s3 Supplemental Table 1. Cardiovascular disease risk factors assessed by cardiology centres.
