# Supplementary material for: Secondary Preventive Care for Cardiovascular Diseases in Bangladesh: A National Survey
Source: Glob Heart. 2021 Apr 30;16(1):31. doi: 10.5334/gh.953 (PMC8086718; doi:10.5334/gh.953)
Supplement: Supplemental Figure 1. — Flow diagram depicting identification of cardiac centres and response rate. [file gh-16-1-953-s1.pdf]

Supplemental Figure 1 Flow diagram depicting identification of cardiac centres and response rate

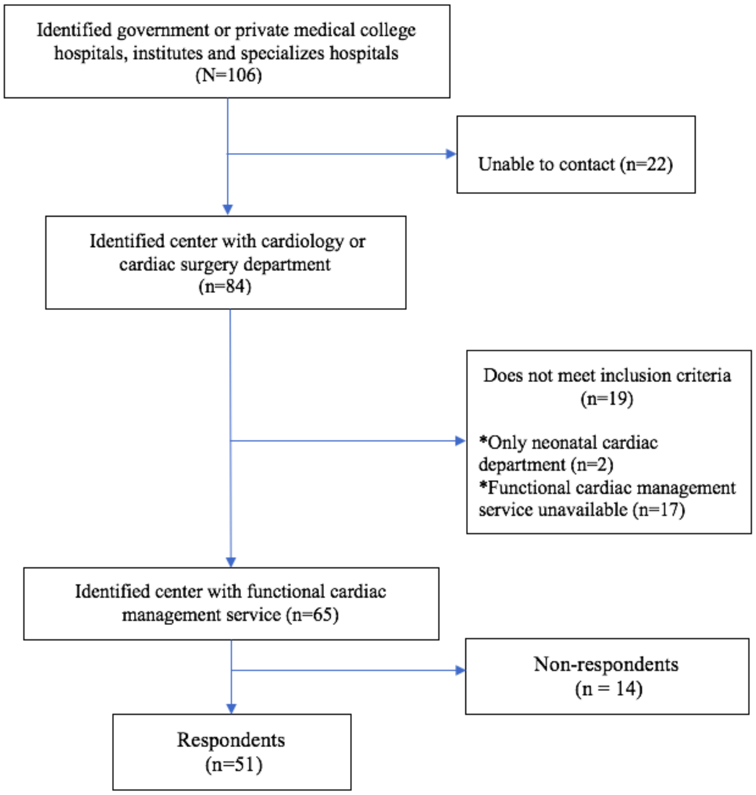

Supplemental Figure 2: Responding center location by Bangladesh district

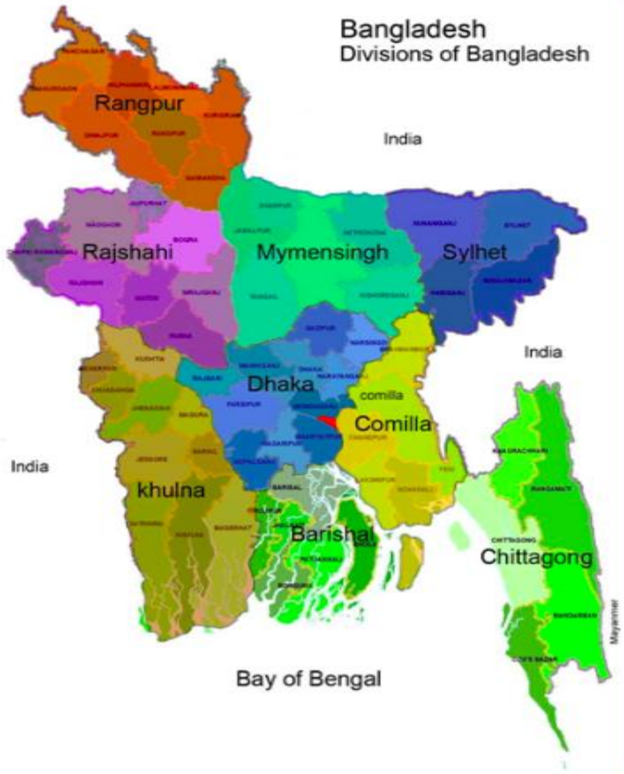

Bogra
Chittagong
Comilla (n=2)
Coxsbazar
Dhaka (n=26)
Dinajpur (n=2)
Faridpur
Gazipur
Golpalgonj
Khulna (n=3)
Kishoreganj(n=2)
Manikganj
Mymensingh
Noakhali
Rajshahi
Rangpur (n=2)
Shirajganj
Sylhet
Tangail
Jessore

Supplemental Table 1. Cardiovascular disease risk factors assessed by cardiology centres

	N	%
Blood pressure	51	100.0
Total Cholesterol	51	100.0
Tobacco use	50	98.0
HDL, LDL Cholesterol	50	98.0
Triglycerides	50	98.0
HbA1c for diabetic patients	49	96.0
Harmful use of alcohol	47	92.1
Time spent being sedentary	45	88.2
Physical inactivity	44	86.0
Body mass index	43	84.3
Poor diet	42	82.3
Blood glucose for non-diabetic patients	40	78.4
Depression / anxiety	32	62.7
Waist circumference	17	33.3
Sleep apnea	14	27.4
Body composition	12	23.5
Other risk factor(s)	0	0.0

Abbreviation: N= Number, %= Percentage.
HDL= High density lipoprotein, LDL= Low density lipoprotein
