# Supplementary figures and images for: Secondary Preventive Care for Cardiovascular Diseases in Bangladesh: A National Survey
Source: Glob Heart. 2021 Apr 30;16(1):31. doi: 10.5334/gh.953 (PMC8086718; doi:10.5334/gh.953)

Supplemental Figure 2: Responding center location by Bangladesh district

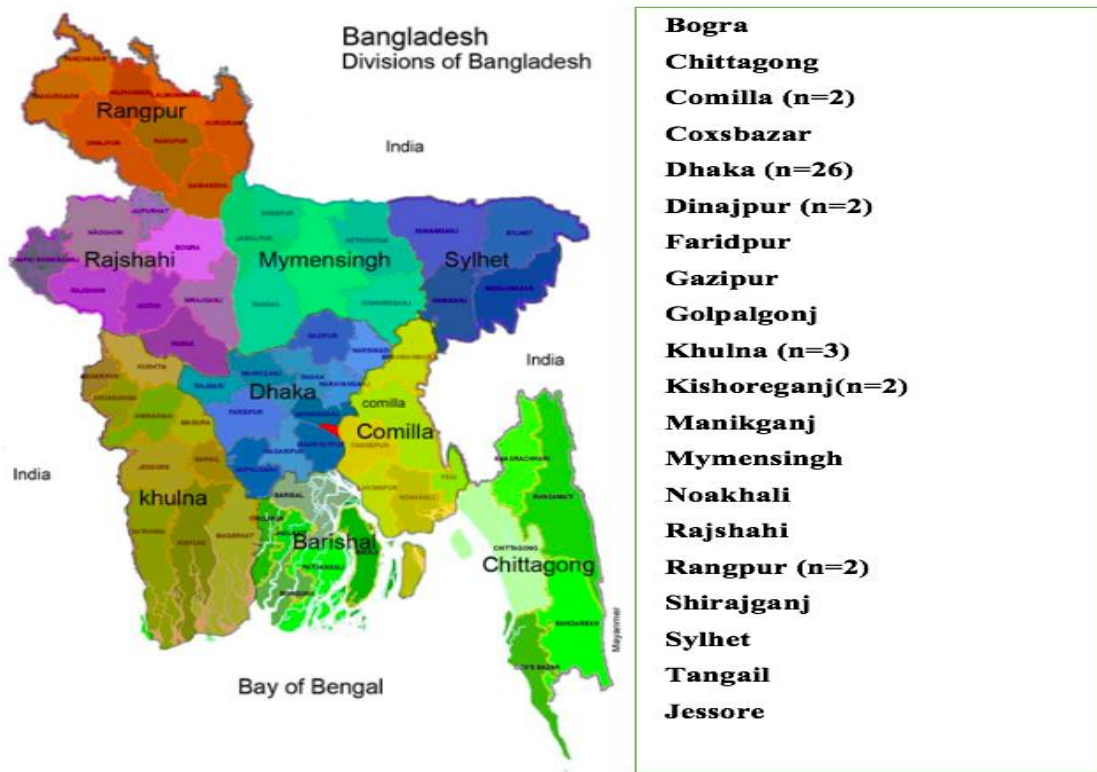

Supplement: Supplemental Figure 2. — Responding center location by Bangladesh district. [file gh-16-1-953-s2.pdf]
